# Automated microfluidic platform of bead-based electrochemical immunosensor integrated with bioreactor for continual monitoring of cell secreted biomarkers

**DOI:** 10.1038/srep24598

**Published:** 2016-04-21

**Authors:** Reza Riahi, Seyed Ali Mousavi Shaegh, Masoumeh Ghaderi, Yu Shrike Zhang, Su Ryon Shin, Julio Aleman, Solange Massa, Duckjin Kim, Mehmet Remzi Dokmeci, Ali Khademhosseini

**Affiliations:** 1 Harvard-MIT Division of Health Sciences and Technology, Massachusetts Institute of Technology, Cambridge, MA 02139, USA; 2 Biomaterials Innovation Research Center, Department of Medicine, Brigham and Women’s Hospital, Harvard Medical School, Cambridge, MA 02139, USA; 3 Wyss Institute for Biologically Inspired Engineering, Harvard University, Boston, MA 02115 USA; 4 Department of Physics, King Abdulaziz University, Jeddah 21569, Saudi Arabia; 5 College of Animal Bioscience and Technology, Department of Bioindustrial Technologies, Konkuk University, Hwayang-dong, Kwangjin-gu, Seoul 143-701, Republic of Korea

## Abstract

There is an increasing interest in developing microfluidic bioreactors and organs-on-a-chip platforms combined with sensing capabilities for continual monitoring of cell-secreted biomarkers. Conventional approaches such as ELISA and mass spectroscopy cannot satisfy the needs of continual monitoring as they are labor-intensive and not easily integrable with low-volume bioreactors. This paper reports on the development of an automated microfluidic bead-based electrochemical immunosensor for in-line measurement of cell-secreted biomarkers. For the operation of the multi-use immunosensor, disposable magnetic microbeads were used to immobilize biomarker-recognition molecules. Microvalves were further integrated in the microfluidic immunosensor chip to achieve programmable operations of the immunoassay including bead loading and unloading, binding, washing, and electrochemical sensing. The platform allowed convenient integration of the immunosensor with liver-on-chips to carry out continual quantification of biomarkers secreted from hepatocytes. Transferrin and albumin productions were monitored during a 5-day hepatotoxicity assessment in which human primary hepatocytes cultured in the bioreactor were treated with acetaminophen. Taken together, our unique microfluidic immunosensor provides a new platform for in-line detection of biomarkers in low volumes and long-term *in vitro* assessments of cellular functions in microfluidic bioreactors and organs-on-chips.

Microfluidic bioreactors and organs-on-chips are emerging devices to model physiological functions of tissues and organs in a controlled environment^1^. These devices produce biomimetic units that can recapitulate tissue- and organ-level functions for applications such as disease modeling, and pre-clinical *in vitro* drug screening^1^,^2^,^3^,^4^,^5^,^6^. In order to perform a precise analysis, often alteration of cellular behavior and response to the changes in the microenvironment should be continuously monitored, ideally using integrated in-line sensors^1^. Such sensors should be capable of continuous measurement of single or multiple analytes in small sample volumes for extended periods of time particularly for applications that deal with chronic or retarded cellular reactions to certain drugs or conditions. In addition, a sensing platform should allow for convenient integration with a microfluidic bioreactor with the capability of automated interface and integrated control^7^, which can improve the accuracy of measurements. Conventional approaches such as ELISA and mass spectroscopy cannot fulfill the requirements of continual monitoring because they are labor-intensive and not easily integrable with low-volume bioreactors. Thus, development of microfluidic platforms with integrated in-line sensing capabilities for long-term continual analysis plays a vital role in the advancement of organs-on-chip devices for *in vitro* assessments of cellular functions.

Integration of analytical detection methods with microfluidics can potentially improve the detection performance by reducing the analysis time, decreasing the consumption of liquid samples and reagents, and increasing reliability through standardization and automation^8^,^9^. Among the available analytical methods integrated with microscale systems to measure biomolecules^10^,^11^,^12^,^13^,^14^,^15^,^16^,^17^,^18^,^19^, electrochemical (EC) techniques^20^,^21^,^22^,^23^ are highly suited for microfluidic systems. This is mainly due to easy miniaturization of detection elements and high degree of integration ability with analytical functions for analysis of small-volume biochemical samples at low cost^22^. These features of EC methods allow for fabrication of compact sensing platforms that are capable of continuously detecting biomolecules with high selectivity, as well as, convenient access by end users. In particular, EC immunosensors for bimolecular detection or kinetic analysis take advantage of the high sensitivity and specificity of the antibody-antigen interactions, whereby antibodies are usually immobilized on the surface of the EC electrode for antigen detection^8^,^22^. However, such a surface immobilization strategy hinders the use of the sensor for continual analysis of biomolecules due to the saturation of the electrodes over repeated detection cycles^23^.

An approach to construct multi-use EC immunosensors relies on the use of disposable microbeads to immobilize antigen-recognition molecules^24^. Once a cycle of measurement is completed, the microbeads can be replaced with new ones. This approach enables the microelectrode to be used repeatedly over many measurements. Microbeads provide a large surface area to immobilize recognition molecules in large quantities, which is conveniently modulated by the number of microbeads introduced into the system. In particular, magnetic microbeads (MBs) can be efficiently manipulated in a microfluidic chip in the presence of an external magnetic field and easily flushed out by the liquid flow upon deactivation of the magnet^25^. These features provide necessary capabilities to realize microfluidic MBs-based immunosensors in combination with EC detection methods for biomarker detections^8^ such as Immunoglobulin G (IgG)^26^,^27^, cancer biomarkers^28^, and bacteriophage MS2^29^.

Here, we introduce a MBs-based EC immunosensor contained in a microfluidic perfusion circuitry integrated with a microfluidic liver bioreactor for continual on-chip detection of cell-secreted biomarkers (Fig. 1). Initially, an off-chip arrangement using a well plate was conducted to optimize different experimental parameters affecting the sensitivity of the EC immunoassay. The immunoassay was then implemented in a microfluidic chip with integrated microvalves for automated on-chip detection of a target biomarker (Fig. 1a,b). On-chip manipulation of the required aqueous reagents for biomarker detection was carried out using an automated valve controller system. The microfluidic sensing platform could accurately perform loading and unloading of the MBs, liquid handling for antibody-antigen interactions, covalent binding of streptavidin horseradish peroxidase (SA-HRP) to secondary antibody, as well as sample extraction from the bioreactor. The sensing platform was characterized against HepG2 liver bioreactors for the wide range detection of transferrin (TF) as a known liver biomarker^30^. This protein is mainly synthesized in the liver and it has a critical role to transport and deliver iron in various metabolic processes. The sensing system was further fully integrated with the microfluidic bioreactor containing bioprinted primary human hepatocyte spheroids (Figs 1c and S1), demonstrating its capability of long-term continual monitoring of changes in the level of secreted liver biomarkers including TF and albumin (ALB). Finally, drug toxicity assessment was conducted using this integrated system by monitoring the effect of different acetaminophen (APAP) concentrations on the production rate of the cell-secreted biomarkers.

## Results and Discussion

### Optimization of the bead-based sensing method

Prior to conducting on-chip detection of cell-secreted biomarkers, the immunosensing assay was characterized and optimized off-chip using standard biomarker solutions based on a chessboard titration method. The effect of operating parameters including the number of MBs as well as amount of secondary antibody and SA-HRP on the overall assay time was investigated, as indicated in Fig. 2. The effect of each parameter was evaluated one at a time, and the optimized parameter was adopted for the subsequent experiments. Initially, the quantity of the primary antibody-coated MBs was optimized in the range of 500 to 10,000 beads. Other parameters, including the concentration of the secondary antibody, concentration of SA-HRP, and the incubation time were maintained constant at 2.5 μg/mL, 1 μg/mL, and 30 min, respectively. Incubation process was required for each of the following binding steps: (1) capturing of the target biomarker (antigen) by primary antibody-coated MBs, (2) binding of the secondary antibody to the biomarker, and (3) attachment of SA-HRP to the biotinylated secondary antibody residing on the MBs. The required time for HRP-catalyzed TMB oxidation was fixed at 15 min. TF with a concentration of 4,000 ng/mL was used as a standard solution for all amperometric optimization measurements to cover the wide physiological range.

Figure 2a illustrates the measured current corresponding to TF detection at different quantities of captured MBs obtained at −100 mV applied potential. While the EC signal improved by 40% as the number of beads increased from 5,000 to 7,500 beads, non-significant signal enhancement was observed for subsequent increase in the number of beads. Thus, the number of 7,500 beads was selected for the following experiments. As shown in Fig. 2b, the current response as a function of the secondary antibody concentration was studied within the range of 1.0–10.0 μg/mL. When the concentration of the secondary antibody was increased from 1.0 μg/mL to 2.5 μg/mL, the signal improved more than 1.6 fold from 0.43 μA to 0.71 μA. However, a further increase in antibody concentration resulted in negligible signal improvements. In addition, at concentrations above 2.5 μg/mL, the deviation of the signal response from the average value was large due to non-specific protein binding to the channel surface. Consequently, to reduce the overall operation cost of the sensor associated with the amount of the antibody and maintain a high signal-to-noise ratio, a detection antibody concentration of 2.5 μg/mL was selected as the optimal value for further experiments. The EC immunosensor was subsequently characterized to find a proper value for SA-HRP concentration and to determine the lowest limit of detection. As shown in Fig. 2c, current response was measured using SA-HRP within the range of 0.01–10.0 μg/mL. As the concentration of SA-HRP was increased from 1.0 μg/mL to 10.0 μg/mL, the signal response showed only 15% improvement, whereas, there was a pronounced increase in signal intensity between 0.01 μg/mL and 1.0 μg/mL. More importantly, the increase in the sensor response at 10.0 μg/mL was accompanied by a large deviation from the average signal value causing low assay sensitivity. Thus, a concentration of 1.0 μg/mL was selected as the optimal value for SA-HRP. In addition to optimization of reaction parameters, the effect of incubation time on the amperometric current response was evaluated. Figure 2d revealed that the incubation time affected both the sensor sensitivity and the overall required time to perform the EC immunoassay. A significant rise in current signal was observed when the incubation time was increased from 15 to 30 min; however, longer incubations did not have further significant impact on the measured signal. Thus a 30 min incubation time was selected for each binding step, providing a total of 90-min incubation period. Together, the optimal process for the EC immunosensor consisted of using 7,500 MBs for immobilizing 2.5 μg/mL of detection antibody in combination with 1.0 μg/mL of SA-HRP. A 30-min incubation time was considered sufficient for each binding step at room temperature while 15 min was used as a required time for completion of HRP-catalyzed enzymatic reactions for TMB oxidation.

### Off-chip biomarker detection

The performance of the bead-based immunosensor for detecting TF biomarker was then evaluated off-chip and results were compared with a commercial ELISA kit. Employing optimal conditions, the amperometric measurements were initially carried out using standard TF solutions over the concentration range of 0–16,000 ng/mL (Fig. 3a) to obtain a calibration curve. The amperometric current response reached a plateau within 5 s. Absolute current values increased as a function of increasing oxidized-TMB concentration. Figure 3b illustrates a semi-log calibration curve using the current values obtained at the 5^th^ second of the amperometric measurement. A linear curve was fitted to the data using a regression analysis for the TF concentrations in the linear range between 10 and 4,000 ng/mL (inset). The regression equation was y = 0.079(x) + 0.044, where y signified the current and x was the TF concentration (R^2^ = 0.99). Results revealed that the average relative standard deviation of the signals in the linear range of TF concentrations was <8% (n = 4), thereby showing a good reproducibility of the EC immunoassay.

To investigate the selectivity of the developed EC immunosensor for TF detection, a mixture of standard TF and ALB at equal concentrations of 1,000 ng/mL was analyzed. As shown in Fig. 3c, the immunosensor could accurately detect TF biomarker using magnetic microbeads conjugated with primary antibody (TF+/MBs) in comparison to bare magnetic microbeads (TF−/MBs) regardless of presence or absence of ALB. Such data demonstrated that the presence of other biomarkers had minimal effect on the detection. For the control test, a cell culture medium with no TF and ALB was measured, and the data showed no significant biomarker presence which was considered as the background noise. Thus results confirmed the reliability and specificity of the developed immunosensor for detection of the target biomarker with statistical significance. After testing the EC immunosensor with standard solutions, we next tested it against real sample solutions extracted from the HepG2 bioreactor. In order to compare the amount of secreted TF at different days, the number of cells cultured in the bioreactor was counted. Initially, 2.0 ± 0.5 × 10^5^ cells were seeded in the bioreactor in which the cell number increased from 2.4 ± 0.4 × 10^5^ cells at Day 1 to 3.6 ± 0.5 × 10^5^ cells at Day 5. As shown in Fig. S2a, a strong signal corresponding to the TF secretion rate of 5.7 ± 2.1 μg/day/10^6^ cells was measured for the Day 1 bioreactor sample using the EC immunosensor with TF+/MB. The result was comparable with our previously reported hepatic models in which the TF production rate at Day 1 varied between 7.3 and 12.7 μg/day/10^6^ cells^31^. During the analysis of TF-free culture medium sample, the EC immunosensor using TF+/MB produced no significant signal. Furthermore, the bioreactor sample was analyzed for TF using the EC immunosensor without antibody-coupled MBs. The measurement showed a TF level similar to the background noise. These data confirmed the ability of the bead-based EC immunosensor to detect biological samples, and the signal amplitude was approximately three orders of magnitude higher than the background noise.

In order to confirm the accuracy of the bead-based EC immunosensor, the TF detection results of the HepG2 bioreactor sample at Days 1, 3, and 5 were compared with those from ELISA. The ELISA experiment was performed according to the manufacturer’s protocol and results were analyzed with the plotted semi-log calibration curve (Fig. S3). As shown in Fig. 3d, the results obtained by the EC immunosensor were comparable with the measurements by ELISA for TF detection. As expected, the concentration of TF secreted by the HepG2 cells increased over time. This indicates that we can continually track changes in biomarker concentrations using small liquid volumes. However, EC measurements depicted lower average values with slightly larger deviations in comparison with ELISA results. This characteristic of the EC sensor could be associated with possible loss of MBs throughout the manual operation of the immunoassay, or interferences occurring during manual handling of the solutions for EC measurements^32^.

To investigate the ability of the EC sensor to perform repeated measurements and assess potential structural defects of the sensor over time, three sample solutions were extracted from a bioreactor at Days 1, 3, and 5. Analysis of TF for each extracted sample was repeated at four individual days at two-day intervals using the same fabricated EC sensor (Fig. S2b). Results revealed consistent values with no significant difference between measurements even after repeatedly using the EC sensor for 6 days. Such results indicate the minimal surface and structural defect of the EC sensor reused during the present study. Importantly, the developed EC immunosensor could measure average TF concentrations of 5.8, 10.8, and 14.5 μg/day/10^6^ cells for Days 1, 3, and 5, in agreement with the results reported in the literature for HepG2 cells (0.1–17.2 μg/day/10^6^ cells)^33^,^34^,^35^,^36^. Overall, results shown in Figs 3 and S2 demonstrated the capability of the developed EC immunoassay to perform selective and reproducible TF detection, which is critical for in-line detection of a biomarker from bioreactor samples for long-term continual monitoring.

### Characterization of on-chip magnetic bead loading

**Figure 1:**
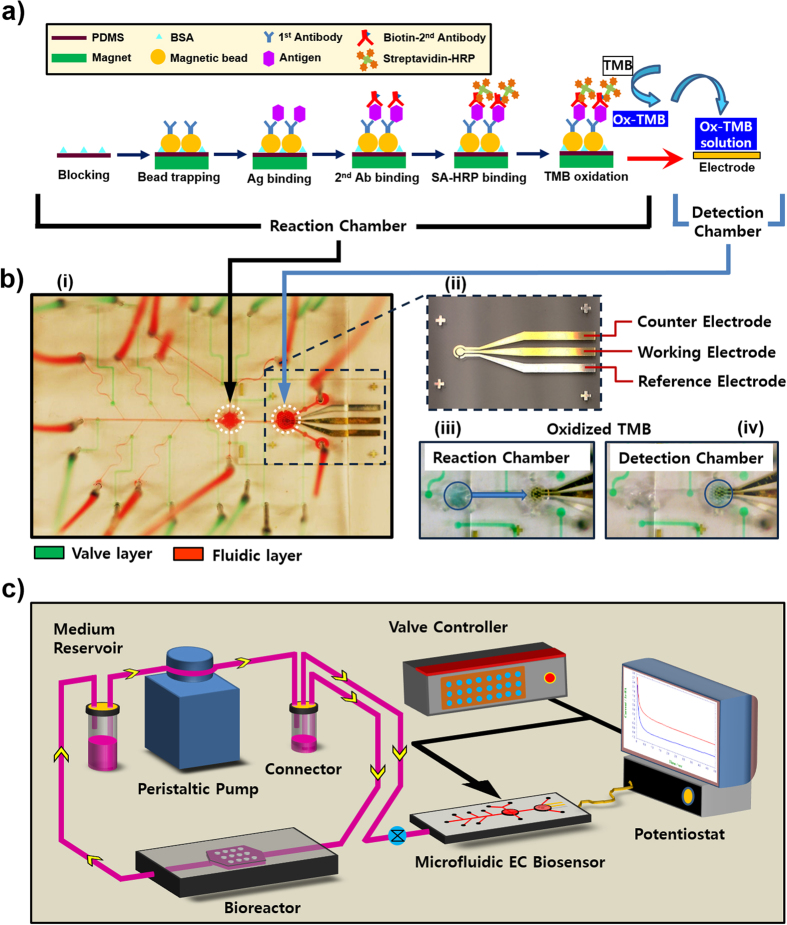
Design, fabrication, and detection principle of the bead-based microfluidic EC sensing system. (**a**) Illustration of the immunosensing principle with the EC sensor for detection of target biomarkers. (**b**) Configuration of the fabricated microfluidic sensing chip, (i) photograph of the microfluidic chip with an integrated microelectrode, (ii) photograph of the microelectrode, (iii) photograph of the reaction chamber with oxidized TMB, (iv) transfer of oxidized TMB to the detection chamber. (**c**) Schematic of the microfluidic sensing system integrated with organs-on-chip for continual monitoring of a target biomarker.

**Figure 2:**
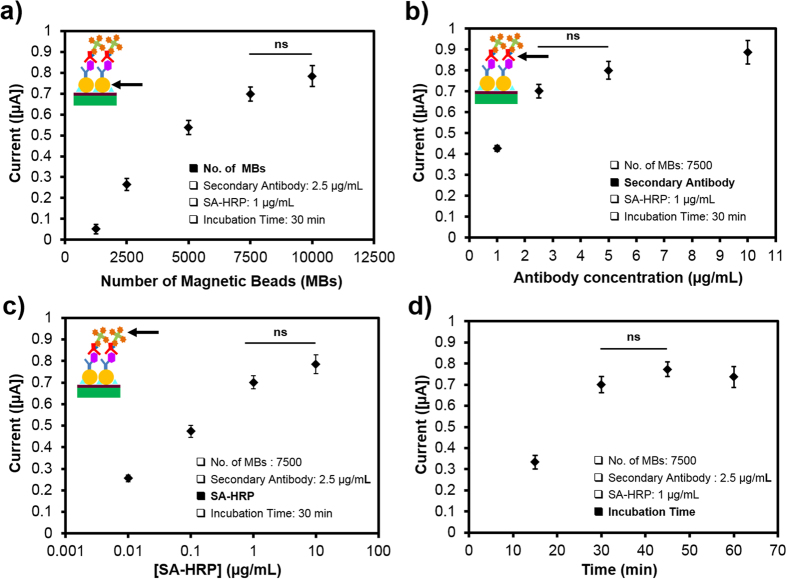
Optimization of the bead-based immunoassay for biomarker EC detection using 4,000 ng/mL TF concentration. (**a**) The number of trapped MBs in the reaction chamber. Effect of (**b**) detection antibody concentration, (**c**) SA-HRP, and (**d**) incubation time on antigen detection while all other parameters were fixed. Data are representative of three independent experiments (ns = not significant).

**Figure 3:**
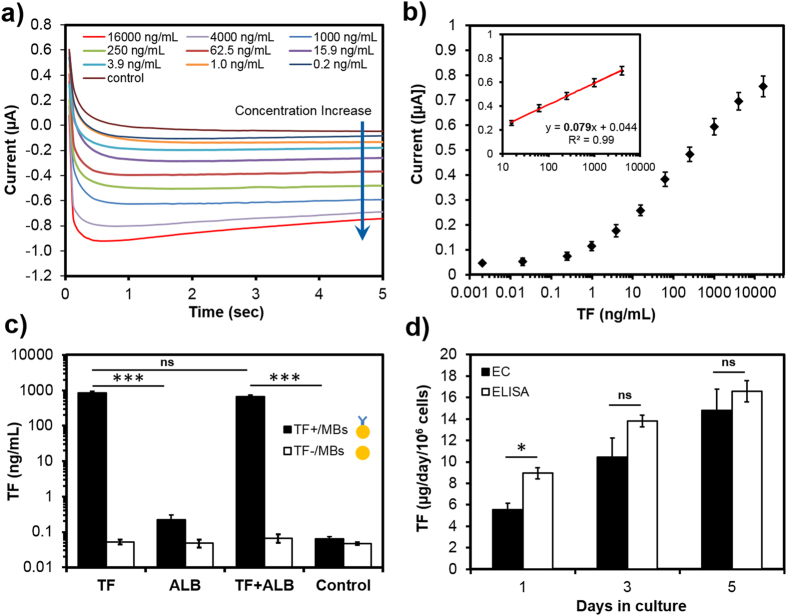
Off-chip TF measurement using the developed EC bead-based immunosensing approach. (**a**) Amperometry measurement of the EC sensor at −100 mV for different standard TF concentrations obtained by 1:4 serial dilutions. (**b**) EC calibration curve for TF obtained from standard TF solutions (0–16,000 ng/mL), insert is linear regression. (**c**) The selectivity of the developed EC immunosensor using MBs with and without coupled TF primary antibody. (**d**) Production rate of TF in HepG2 bioreactor samples measured by ELISA and the developed electrochemical sensor. Data are representative of three independent experiments (ns = not significant; *P < 0.05; ***P < 0.001).

**Figure 4:**
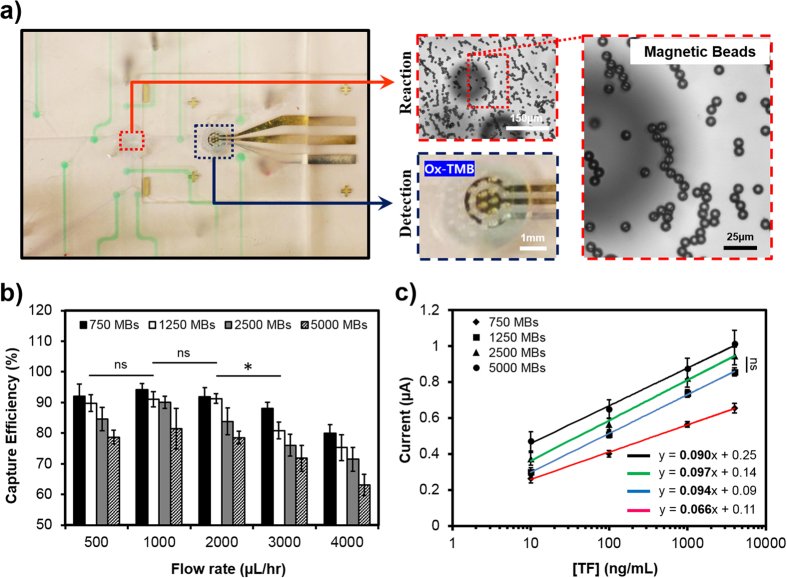
Characterization of on-chip MBs loading. (**a**) Illustration of trapped MBs in the reaction chamber. Circular shadows in the background are bubble trap microposts embedded on the top surface of the reaction chamber. (**b**) On-chip capture efficiency of loaded MBs at different flow rates. (**c**) Sensitivity of the on-chip immunoassay at different numbers of immobilized beads for detection of standard TF concentration in the linear range of the assay (10–4,000 ng/mL). Data are representative of three independent experiments (ns = not significant; *P < 0.05).

To reduce manual errors, consume lower sample volumes, and automate the EC immunoassay for continual analysis of secreted biomarkers, we then developed an on-chip detection platform. To achieve this goal, MBs should be loaded into a reaction chamber implemented in the microfluidic chip (Figs 1b and 4a). In comparison with the off-chip method, the liquid volume of the reaction chamber for on-chip immunosensing was roughly 7 times smaller. Thus, to have a fair comparison between off-chip and on-chip detection methods, the number of immobilized beads was re-optimized. For on-chip immunosensing, the use of a liquid flow at optimal flow rate is required to load and immobilize a certain number of MBs into the reaction chamber (Fig. 4a,b). If the flow rate is too high, the loss of MBs during loading step could result in irreproducible measurements. On the contrary, low flow rate can induce bead retention in the bead loading channel causing inefficient capture of MBs in the reaction chamber^29^. To find the proper flow conditions for bead loading, a range of MBs (500–5,000 beads) from a stock solution (2 × 10^4^ beads/mL) were introduced into the reaction chamber using different flow rates. An external magnet was placed underneath the reaction chamber to immobilize the MBs. Once the bead loading and immobilizing steps were completed, the trapped MBs were flushed out from the reaction chamber and collected in an off-chip reservoir. To examine the capture efficiency, the recovered MBs were counted using a microscope. Figure 4b shows the capture efficiency of the MBs as a function of bead number and liquid flow rate. No significant loss of beads was observed during loading and recovery of 750 and 1,250 MBs using flow rates between 500–2,000 μL/hr. Nevertheless, at higher flow rates the capture efficiency for all loaded beads decreased, potentially impacting the reproducibility of the sensing assay. Therefore, a flow rate of 2,000 μL/hr was selected as an optimal value for bead loading.

To find the optimal quantity of required immobilized beads for on-chip measurements, the TF detection with the standard concentration of 10–4,000 ng/mL (the linear range of the assay) was examined for various numbers of loaded MBs (750–5,000). As shown in Fig. 4c, measured signals for different numbers of loaded beads were fitted with linear curves using regression analysis. The slope of the curve, representing the sensor sensitivity, was affected by the number of MBs. The largest slope was achieved using 2,500 MBs. However, when the bead number was reduced from 2,500 to 1,250, no significant loss of signal and sensitivity were observed over the range of target concentrations. This observation could be due to the lower capture efficiency (~83%) with higher deviation for 2,500 MBs at the flow rate of 2,000 μL/hr compared to 1,250 MBs (Fig. 4b,c). In addition, 1,250 MBs showed minimal signal variation for detection of a target molecule, which is an indication of enhanced assay reproducibility. Thus, 1,250 MBs were selected for on-chip testing to ensure the high assay sensitivity and reproducibility. Interestingly, this number of MBs chosen for optimization of on-chip detection was almost 6 times less than the number used for off-chip measurement, which correlated with the 7-to-1 ratio between the off-chip and on-chip reaction chamber volumes mentioned earlier.

Together, our results demonstrated that the capture efficiency was a function of the flow rate at which the MBs were introduced into the reaction chamber and the number of the MBs in solution. Furthermore, it was revealed that the optimal number of loaded MBs for the on-chip EC measurements was achieved under a compromise between high sensitivity and high reproducibility of the immunoassay, which is consistent with the available literature^29^. Importantly, the efficient bead loading and recovery of the developed system with the capability of repeatedly replacing used MBs with new MBs provides a multi-use on-chip platform for continual biomarker detection.

### On-chip biomarker detection

Using the optimized operating conditions, a microfluidic bead-based EC immunosensor with programmed operation was implemented for the detection of TF biomarker in sample solutions. The on-chip immunoassay was realized using N_2_ gas-actuated microvalves for manipulating the injection of required reagents and samples^37^. Operation of the microvalves was controlled using a WAGO controller^38^ with custom-written MATLAB codes. Using this control system, liquid injections from individual reservoirs were accomplished for accurate flow manipulation within the chambers. To start the automated detection process, 1,250 MBs conjugated with TF primary antibodies were introduced into the chip and trapped within the reaction chamber at a preset flow rate of 2,000 μL/hr. All binding steps and enzymatic reactions were performed inside the reaction chamber. Subsequently, oxidized TMB was introduced into the detection chamber for analysis of TF concentrations using the integrated microelectrode. In this configuration we separated the reaction chamber from the detection chamber and this design enabled us to minimize the effect of different binding processes on sensing electrodes and allowed us to obtain consistent EC measurements of the target biomarker concentration regardless of the culture medium used (Fig. S4).

**Figure 5:**
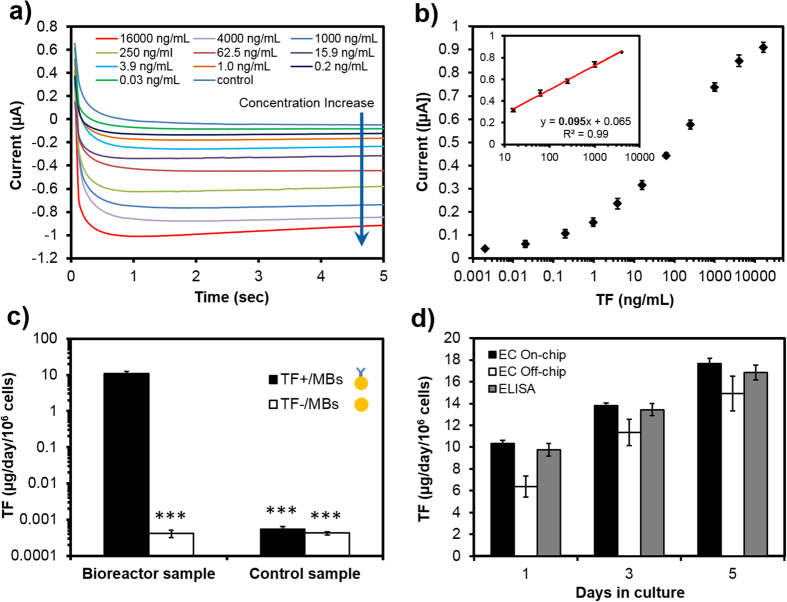
On-chip detection of bioreactor samples. (**a**) Amperometry measurement at −100 mV for different standard TF concentrations obtained by 1:4 serial dilutions. (**b**) Corresponding EC calibration curve for TF biomarker. (**c**) On-chip measurement of TF in bioreactor samples obtained from Day 1. (**d**) Comparison between on-chip and off-chip measurements of TF production rate in HepG2 bioreactor samples obtained from Day 1, 3, and 5. Data are representative of three independent experiments.

Following the immunoassay protocol described in the Methods section, standard biomarker solutions were analyzed over a wide range of TF concentrations (0–16,000 ng/mL). Similar to the off-chip method, the absolute value of current signal was elevated as the concentration of TF was increased (Fig. 5a). Using the signal values obtained at the 5^th^ second of each measurement, a semi-log calibration curve was plotted and a linear curve, y = 0.095(x) + 0.065, was obtained for the TF concentrations between 10–4,000 ng/mL (Fig. 5b). It appeared that the linear portion of semi-log calibration curve had a higher slope compared to the off-chip method, demonstrating a higher sensitivity for the automated on-chip immunoassay presumably due to the automated handling and manipulation of liquids in the microfluidic chip. In addition, the on-chip measurement had a limit of detection (LOD) of 0.03 ng/mL, which was almost one order of magnitude better than the one obtained from off-chip and ELISA tests (0.2 ng/mL).

To evaluate the selectivity of the EC on-chip immunosensor for the measurement of TF in bioreactor samples, two sets of experiments were executed on-chip using TF+/MBs and TF−/MBs. Liquid samples were extracted from the microfluidic bioreactor, containing HepG2 cells, at Day 1, and the cell culture medium was used as a control sample. HepG2 human hepatocarcinoma cells are usually employed as a liver cell model in microfluidic bioreactors^39^. As shown in Fig. 5c, the on-chip immunosensor was able to detect a TF biomarker concentration of 10.8 ± 0.9 μg/day/10^6^ cells, while the response signal from the EC sensor using TF−/MBs was negligible. Also, the control sample showed non-significant measured value of TF biomarker in the cell culture medium. Such data confirmed the ability of the developed microfluidic immunosensor for selective detection of TF from the biological sample.

In another series of experiments, the results obtained from the microfluidic bead-based EC immunosensor (on-chip method) were compared with the results from off-chip and ELISA measurements using HepG2 bioreactor samples at Days 1, 3, and 5 in culture (Fig. 5d). The comparison revealed that the on-chip measurements were in agreement with off-chip and ELISA data. TF average values measured by the on-chip immunosensor were 10.34, 13.8, and 17.6 μg/day/10^6^ cells while the corresponding results obtained from ELISA kit were 9.7, 13.4, and 16.8 μg/day/10^6^ cells at Days 1, 3 and 5, respectively. However, on-chip detection showed smaller deviations for each sample compared with both off-chip and ELISA measurements. It also appeared that on-chip data were more consistent with the results obtained by the commercial ELISA kit compared with off-chip measurements. Such accurate measurement proved the reliability of the on-chip immunoassay for detection of the target biomarker.

Overall, the concentration values measured by the on-chip method were within the range of 9–18 μg/day/10^6^ cells that were comparable to the values reported in the literature for TF secreted by HepG2 cells^33^,^34^,^35^. The integrated on-chip immunosensor demonstrated a higher sensitivity and a lower limit of detection in comparison with the off-chip system. Such efficient performance of the on-chip method is mainly attributed to its programmed operation for MBs loading, sample handling, and signal reading. This on-chip detection provided reproducible detections by eliminating possible human errors. In addition, a lower number of beads were used for on-chip detections that produced more consistent measurements. Besides, small volume of the reaction chamber provided a shorter diffusion length to improve antibody-antigen interactions^40^. Importantly, the small amounts of biological samples and reagents used for on-chip analysis reduced the cost of operation and the assay time. Therefore, the on-chip immunoassay has provided a reliable and cost-effective detection method for biomarker measurements in biological samples.

### Continual monitoring of cell-secreted biomarkers from an integrated bioreactor

**Figure 6:**
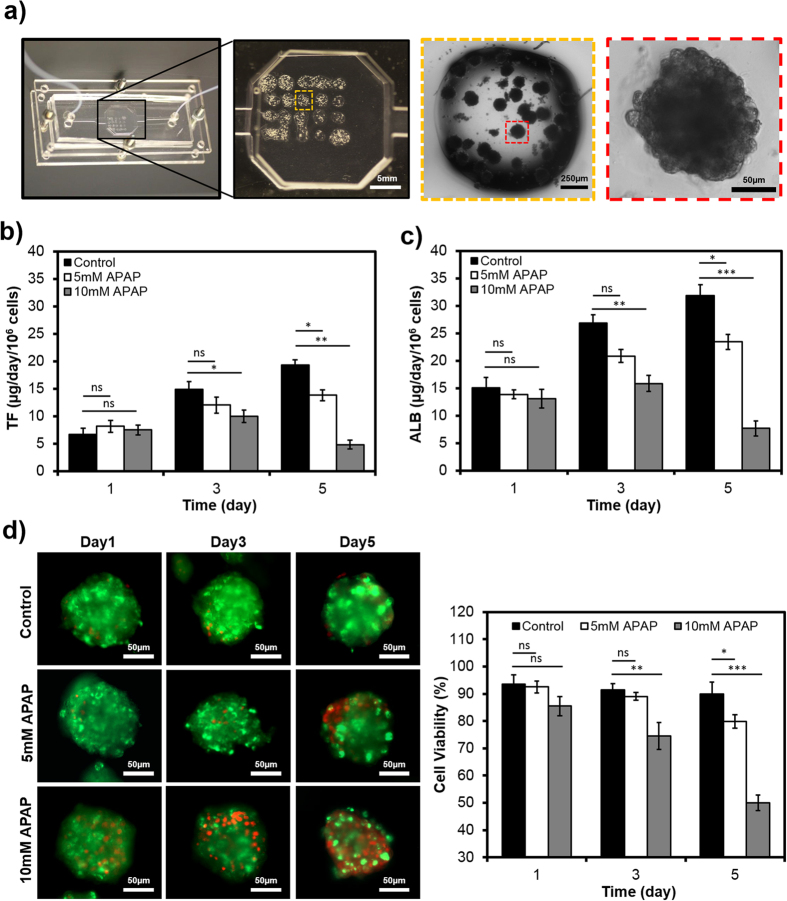
On-line measurement of biomarkers secretion from primary hepatocyte bioreactor construct upon drug treatment. (**a**) Photograph of the fabricated bioreactor with bioprinted primary hepatocyte spheroids. (**b,c**) Continual EC measurements of TF (**b**) and ALB (**c**) production rate in primary hepatocyte bioreactor with 5 mM and 10 mM APAP treatment. (**d**) Live/dead staining and cell viability quantification of primary hepatocyte spheroids treated with 5 mM, and 10 mM APAP. Staining was performed at Day 1, 3, and 5 using calcin AM (green) and ethidium homodimer (red). Data are representative of three independent experiments (ns = not significant; *P < 0.05; **P < 0.01; ***P < 0.001).

We next analyzed the performance of our bead-based immunosensor for its capability in continually monitoring cell-secreted biomarkers in a microfluidic bioreactor. Microfluidic bioreactors and organs-on-chip devices are recently-developed microsystems that recapitulate the biology and physiology of human organs on an integrated microfluidic circuitry for disease modeling and drug and toxin screening^5^,^6^,^41^. Particularly, we realized a microfluidic platform consisting of an *in vitro* hepatotoxicity model in a microscale bioreactor combined with the microfluidic immunosensor to study the change in the levels of biomarkers secreted from drug treated human liver cells (Figs 1c and S1). The sensing platform was directly connected to the developed bioreactor including bioprinted human primary hepatocyte spheroids (Fig. 6a), which were constructed from 2.5 ± 0.4 × 10^5^ cells. We administered acetaminophen (APAP) to the bioreactors at different concentrations to monitor protein secretion. It is well known that exposure of hepatocytes to APAP can alter the rate of protein secretion mediated by metabolic activities^42^. In this study, 5 mM and 10 mM APAP were selected based on the lethal concentration 50 (LC50) values previously optimized by us and available in the literature for hepatotoxicity studies^31^,^43^. To preserve liver metabolic activity, the bioreactor was placed under a continuous perfusion of the hepatocyte culture medium at a flow rate of 200 μL/hr using a peristaltic pump in a closed-loop arrangement (Fig. S1). During the detection process, sample solutions were transferred from the bioreactor into the sensing platform through a programmable microvalve at desired intervals, and amperometry measurements of TF and ALB biomarkers were performed at Days 1, 3, and 5 of culture (Fig. S5a,b). Figure S5c,d represent the current signal corresponding to different TF and ALB biomarker concentrations in bioreactor samples obtained at different days upon drug treatments. The actual amounts of the biomarkers were then obtained using their respective calibration curves (Figs 5b and S6).

Figure 6b illustrates the changes in the level of secreted TF upon drug treatment. The production rate of TF increased from Day 1 through Day 5 for the bioreactor without APAP treatment (control). When the cells were treated with 5-mM APAP, the production rates of TF at Days 1 and 3 were not statistically different from the control indicating the minimal effect of this concentration on cultured cells in the bioreactor after 3 days. In addition, at the 5-mM APAP concentration the change in TF secretion rate from Day 3 to Day 5 was also negligible. However, for 10 mM drug treatment, the production of the biomarker was significantly lower at Day 3 and Day 5 in comparison with the control (Fig. 6b). Similar observations were also made for the ALB biomarker (Fig. 6c); nevertheless, the production rate of ALB was higher than TF through the 5-day assessment regardless of drug concentrations. Meanwhile the average production rate of TF for untreated cells increased from 6.7 μg/day/10^6^ cells at Day 1 to 19.3 μg/day/10^6^ cells at Day 5, The ALB production rate improved from 15.1 μg/day/10^6^ cells at Day 1 to 31.8 μg/day/10^6^ cells at Day 5. These observations were in agreement with the reported results for TF (3–18 μg/day/10^6^ cells) and ALB (6–40 μg/day/10^6^ cells) secreted by human primary hepatocytes within the first 5 days in culture^44^,^45^,^46^,^47^,^48^.

In order to have a qualitative comparison between the measured values for cell-secreted biomarkers and the number of viable cells, we further performed cell viability assay at different APAP concentrations. As shown in Fig. 6d, the bioreactor without APAP treatment (control) showed high cell viability throughout the experiments. Minimal cell death was also observed for the bioreactor treated with 5 mM APAP, which showed ~80% viability at Day 5. However, for the group treated with 10 mM APAP the cell viability dropped significantly at Days 3 and 5, indicating the pronounced cytotoxicity of the drug at such an elevated concentration of APAP. Remarkably, our data showed that the trend in cell viability at each day (Fig. 6d) was consistent with the variation of TF and ALB production rates of the same time points (Fig. 6b,c). This observation is also in agreement with hepatotoxicity studies reporting the impact of APAP on cell viability and rate of biomarker secretion^31^,^49^.

## Conclusions

We have designed, fabricated, and characterized a new programmable microfluidic bead-based EC immunosensor. The sensitivity of the EC immunoassay was optimized off-chip against the number of MBs, concentrations of secondary antibody and SA-HRP, and the incubation time, after which, the biomarker measurements were performed within the biological concentration range (0–16,000 ng/ml). The automated operation of the microfluidic system for biomarker detection was then achieved through integrated microvalves for required liquid manipulations including bead loading, binding steps, washing processes, and sample loading. Results demonstrated the capability of the EC immunosensor to perform sensitive and reproducible measurements of TF present in sample solutions extracted from a hepatic bioreactor. Importantly, the on-chip EC immunosensor showed a low LOD of 0.03 ng/mL, which was one order of magnitude better than that for ELISA (0.2 ng/mL) measured in this study. This observation is mainly attributed to the programmed operation of the EC microfluidic immunosensing with enhanced mass transport within the reaction and detection chambers implemented on the microfluidic circuitry. When directly integrated with a human primary liver-on-a-chip platform, our automated EC immunosensor was capable of long-term continual measurement of the change in the amount of cell-secreted TF and ALB upon APAP treatment, which correlated well with the ELISA and cytotoxicity analyses.

Our sensing system takes advantage of using MBs for immobilization of the detection biomolecules. This feature prolongs the life span of the microfluidic circuitry and the EC microelectrode, which enables the EC immunosensor to be reusable for multiple measurements by disposing MBs after each biomarker analysis. Alternatively, MBs that are functionalized with a different antigen on the surface can be further employed, resulting in the sensing of multiple biomarkers of interest using the same device on demand. Overall, the developed microfluidic platform provides a powerful tool for continual and long-term drug screening in biomedical applications. With further improvement on multiplexed detection, this system is likely to address the unmet needs to improve the current state of *in vitro* diagnostic technologies in tissue engineering, organs-on-chips, drug discovery, and personalized medicine.

## Material and Methods

### Reagents and solutions

The 6.6-μm carboxylated MBs (HOOC-MBs) and xMAP antibody coupling kit were purchased from Luminex (Austin, TX, USA). Primary antibodies against TF and ALB with corresponding biotin-coupled secondary antibodies were available from Abcam (Cambridge, MA, USA). 3,3′,5,5′-tetramethylbenzidine dihydrochloride (TMB) and hydrogen peroxide (H_2_O_2_) were purchased from Sigma-Aldrich (St. Louis, MO, USA). SA-HRP conjugate to oxidize TMB was obtained from Origene (Rockville, MD, USA). A mixture of 1:1 TMB and H_2_O_2_ substrate solutions was prepared before the experiment and protected from the light. Stock standard solutions of biomarkers were prepared in culture medium using Dulbecco’s Modified Eagle’s Medium (DMEM) obtained from Life Technology (Carlsbad, CA, USA). A 1X phosphate buffered saline (PBS) solution with 0.05% Tween-20 (Sigma-Aldrich) was prepared as a washing buffer. 1% bovine serum albumin (BSA) diluted in 1X PBS was used as a blocking solution and diluent. All other chemicals unless otherwise specified were obtained from Sigma-Aldrich.

### Design and components of the microfluidic bead-based platform

Figures 1 and S7 illustrate working principles and a schematic of the developed bead-based microfluidic system for EC detection. As shown in Fig. 1a, MBs functionalized with either TF or ALB primary antibody are loaded and immobilized in the reaction chamber using a magnet. Then, the sample solution containing the target antigen is introduced into the chip where they are bound to the immobilized primary antibody. The molecules are then bound again with a secondary antibody that can be covalently linked to an enzyme (SA-HRP) to oxidize TMB at the presence of H_2_O_2_. Finally, the oxidized TMB is directed into the detection chamber to be sensed on the electrode surface. The magnitude of the measured signal by the electrode quantifies the amount of the target biomolecule in the sample volume.

The microfluidic immunosensing chip was comprised of three polydimethylsiloxane (PDMS) layers including a fluidic layer and a valve layer, which were separated by a 40-μm-thick PDMS membrane to act as a flexible diaphragm for valving (Fig. S7a)^50^,^51^. The fluidic handling layer consisted of microchannels for introducing and extracting liquids, a reaction chamber for immobilization of MBs with an external magnet and sample preparation, and a detection chamber for the target biomolecule detection. The PDMS membrane was in direct contact between the fluidic and valve layers to act as pneumatic microvalves. All the microvalves in the valve layer were designed in circular shape with a 200-μm diameter so that when bonded at the bottom of the fluidic layer, each valve would completely cover the enlarged area of the corresponding liquid channel with a semi-circular cross-section (Fig. S7b). To reduce the potential introduction of the bubbles into the chambers of fluidic layer, a pillar-based bubble removal system with an array of 200-μm diameter microposts was built in the valve layer (Fig. S7c). These microposts were located on top of the reaction and detection chambers, separated by the PDMS membrane, to trap and remove generated bubbles that could not be directly pushed out by the fluidic flow. Figures 1b and S7d represent the design and photograph of the microfluidic EC immunosensing system in which the fluidic layer and the valve layer are shown in red and green colors respectively.

For the continual measurement of a target biomarker, the fabricated microfluidic EC immunosensing chip was further integrated with a microscale bioreactor as illustrated in Fig. 1c and S1. The bioreactor perfusion was performed in a closed-loop arrangement employing a four-channel precision micro peristaltic pump (Cole Parmer, Vernon Hills, IL, USA) with flow rate set to 200 μL/hr, which allowed a sample to be transferred into the reaction chamber upon request using a microvalve.

### Fabrication of microfluidic chips

Both fluidic layer and valve layer of the bead-based microfluidic immunosensing chip were fabricated using the standard soft lithography replica molding technique. Briefly, each mold was created through a single-layer process using negative photoresist, SU8-2050 (MicroChem, Westborough, MA, USA). A microfeatured master mold was then obtained by contact photolithography. To obtain a PDMS microstructure of fluidic and valve layers, a mixture of prepolymer with curing agent (Sylgard 184, Dow Corning, Midland, MI, USA) were prepared at a 5:1 ratio, degassed in a vacuum chamber for 30 min, and poured on the SU8 master mold. After 2 hr incubation at 80 °C in an oven, the PDMS layers was cured and carefully peeled off from the mold. For further creation of a semi-circular cross section of the fluidic layer, the secondary master mold was constructed (Fig. S7b). The negative pressure was applied to the fabricated PDMS fluidic layer with square-cross-section microchannels and SU8-50 photoresist was loaded on a vacuum-assisting surface to fabricate the new master mold. The structure was maintained for 30 min by UV exposure to harden the mold and generate a layer with the semi-circular cross section. The final microfluidic layer was molded by curing a 10:1 mixture of PDMS prepolymer and curing agent against the secondary SU8 master mold. The PDMS replica of the fluidic layer was placed on top of the semi-cured 40-μm PDMS membrane layer followed with 30-min incubation at 80 °C. The PDMS valve layer was then aligned and plasma bonded onto the membrane-bonded fluidic layer. The entire device was then kept in the oven for another hour to assure tight bonding. The overall dimensions of the bead-based microfluidic immunosensing chip was 53 mm (length) × 24 mm (width). The height of microchannels was 0.15 mm and the diameter of reaction and detection chambers was 7.5 mm, which resulted in a total volume of ~7.0 μL per chamber. Also, the width of the channels in both fluidic and valve layers was 200 μm.

### Microfabrication of detection electrode

To achieve high sensitivity and ease of integration into microfluidic systems, we designed and fabricated an EC miniaturized sensing unit made of three electrodes: reference (RE), counter (CE), and working (WE) electrodes (Fig. S8). In a typical procedure, the first shadow mask was used to successively deposit a 20-nm thick titanium (Ti) layer, a 20-nm thick palladium (Pd) layer, and a 500-nm thick gold (Au) layer onto a glass substrate by electron beam deposition (Denton Vaccum, Moorestown, NJ, USA). The thicknesses of the Ti and Pd layers were chosen according to optimized parameters reported in the literature^52^. In this study 20-nm thickness of the Ti layer was selected to achieve the strong adhesion of the Au layer. Furthermore, microfabricated electrodes were annealed at 300 °C for 6 hr in the furnace to reduce potential structural defects. The 20-nm Pd layer was added between the Au and Ti layer to prevent the inter-diffusion between Ti and Au layer during the annealing process as a barrier layer^53^. In addition, the 500-nm thickness of the Au layer was used to improve the long-term usability of the electrodes. Figure S8d illustrates the Nyquest curve obtained for the 500-nm Au thickness compared to the 150-nm thickness, where 500-nm Au layer followed the expected theoretical shape with lower charge resistance transfer (Rct) value than that of 150-nm one.

The second shadow mask was then used for 500 nm silver (Ag) RE generation. The mask was attached to the Au-deposited glass slide and aligned with the patterns using cross-shaped alignment keys. A 20-nm thick Ti layer as an adhesion layer to the substrate, a 20-nm thick Pd layer as a diffusion barrier, and a 500-nm thick Ag layer were next deposited onto the glass substrate. In order to make the Ag/AgCl reference electrode, the chlorination of the Ag layer was carried out electrochemically in a 0.1-M HCl solution by applying constant current of 10 μA for 5 s. The fabricated three-electrode sensing unit had a 800-μm diameter of working electrode, 150-μm wide counter and reference electrodes, and a spacing of 200 μm between each electrode (Fig. S8a). Figure 1b shows a photograph of the assembled microfluidic chip with the fabricated three-electrode sensing unit plasma bonded to the bottom of the detection chamber.

### Automated biomarker detection

The sample immobilization and analysis process was automated by developing a MATLAB (Mathworks, Natick, MA, USA) program to control a WAGO-based controller. The WAGO controller was combined with solenoid valves from FESTO (Chicago, IL, USA)^38^. The control system was used to conduct controlled operation of liquids’ injection from the reservoirs into the microfluidic chip, and also individual N_2_ gas-actuated microvalves for manipulation of required liquids.

In order to inject a liquid into the microfluidic chip, an air pressure of 0.8 psi was applied to the 1-mL liquid reservoirs using program-controlled solenoid valves. The normally open PDMS microvalves were also controlled by gas outlets from solenoid valves via liquid-filled tubing shown in Fig. 1b with green color. In a typical sensing process, the reaction chamber was used to perform bead trapping and required binding steps for antigen, secondary antibody, and SA-HRP through injecting reagents. Once all binding steps were completed, the TMB-H_2_O_2_ solution was injected to the chamber and incubated with antigen captured MBs for 15 min. The final product of the enzymatic reaction between TMB and SA-HRP, oxidized-TMB, was sent into the detection chamber to measure target biomolecule concentration electrochemically using the microelectrode.

### EC measurement

In this study amperometry measurements were performed to monitor the current signal induced by oxidized TMB and determine the concentration of target biomarker in the sample. A computer controlled EC analyzer potentiostat CHI 660 (CH Instruments, Austin, TX, USA) was used for signal readout at fixed voltage with pulse width of 5 s and sample interval of 0.05 s.

To characterize the Au-based microfabricated electrode, a 20 μL of 50 mM K_3_Fe(CN)_6_ was used as the electrolyte, and an Electrochemical Impedance Spectroscopy (EIS) was measured to provide visual insight into the dynamics of the electrode (Fig. S8b). In addition, changes in current during the oxidation/reduction reaction of K_3_Fe(CN)_6_ were monitored by cyclic voltammetry (CV) measurement at a 100 mV/s scan rate with 10 cycles (Fig. S8c). To determine redox peaks for TMB solution, the CV experiment was carried out, as shown in Fig. S9. This CV curve enabled us to select an appropriate potential to prevent interference of TMB oxidation during sample detection. Based on the CV curve for TMB, amperometric measurement was fixed at −100 mV and the electrocatalytic reduction current was recorded at 5 s in which the HRP redox reaction approximately reached the equilibrium.

### Preparation of primary antibody-coated magnetic bead

MBs were coated with primary antibody against TF and ALB biomarkers using the xMAP antibody coupling protocol provided by Luminex. Briefly, 1 mL of the 6.6-μm HOOC-MBs commercial suspension (2.5 × 10^6^ bead/mL) was transferred into a 1.5-mL Eppendorf tube. MBs were washed three times with activation buffer solution (0.1 M NaH_2_PO_4_, pH 6.5) by stirring the tube for 20 s. Between each step the MBs were immobilized using a magnetic separator purchased from Luminex and the supernatant was discarded. The carboxylic groups of MBs were activated by 20-min incubation in the EDC/sulfo-NHS mixture solution placed on a plate shaker at 30 rpm. The activated MBs were washed twice with 500 μL of the activation buffer and re-suspended in 500 μL of a 10 μg/mL primary antibody against either TF or ALB. The primary antibody was captured onto the activated beads during the 120-min incubation at 25 °C under continuous stirring (30 rpm). Subsequently, the antibody-coupled MBs were washed 3 times with 500 μL of washing buffer (0.05% Tween-20 in 1X PBS, pH 7.4). After the washing step, prepared antibody-coupled MBs were re-suspended in 1 mL washing buffer, protected from light, and stored at 4 °C until use.

### Bioreactor and drug treatment

The microfluidic bioreactor was constructed as previously described^31^. Briefly, either HepG2 cells (HB8065, ATCC, Manassas, VA, USA) or primary human hepatocytes (HUCPI6, Triangle Research Labs, Triangle Research Park, North Carolina, USA) were thawed, counted (8 × 10^6^ cells per vial), and seeded in a PDMS 200-μm multi-well mold. HepG2 spheroids were formed in DMEM culture medium with 10% heat-inactivated fetal bovine serum (FBS), and 1 μg/mL rat tail collagen gel type I obtained from Life Technologies. Spheroid formation media for primary hepatocyte consisted of Hepatocyte Maintenance Medium (Triangle Research Labs) with 1 μg/mL rat tail collagen gel type I. To maintain high cell viability, the culture medium was replaced every 48 hours. At Day 5 the spheroids were harvested from microwells, made of PDMS, and mixed with a hydrogel prepolymer solution including 10% (w/v) custom made gelatin methacryloyl (GelMA) and 0.5% (w/v) 1-[4-(2-hydroxyethoxy)-phenyl]-2-hydroxy-2-methyl-1-propanone (Ciba Specialty Chemicals, Tarrytown, NY, USA) used as photoinitiator (PI). GelMA droplets with approximately 1 mm in size were bioprinted in the microfluidic bioreactor using an Organovo NovoGen MMX bioprinter (San Diego, CA, USA) to form a 5 × 4 microarray (20 drops with 18 average spheroid number). After 17 s of UV light exposure at 850 mW at a distance of 8.5 cm, the GelMA drops were crosslinked to the glass at the bottom of the bioreactor. The bioreactor setup was operated by a peristaltic pump and included a reservoir holding 4 mL basal medium (Figs 1c and S1). In additional, the bioreactor held about 1 mL culture medium and the flow rate was maintained at 200 μL/hr. For off-chip and on-chip biomarker measurements, media supernatant was collected from the bioreactor on Days 1, 3, and 5 of culture. For hepatotoxicity assessment studies, the liver bioreactor was directly integrated with the automated microfluidic bead-based sensing system and the effect of 5 mM and 10 mM APAP were continuously monitored for 5 days and the secreted biomarkers were measured on Days 1, 3, and 5 of culture.

### Off-chip characterization of MBs immunocomplex

The MB-based immunosensor was first characterized and optimized off-chip using a standard biomarker solution. A calibration curve was obtained using 1:4 serial dilutions of the stock standard solution of TF or ALB. Performance of the optimized immunosensor was further analyzed using biomarker samples manually pipetted from the microfluidic bioreactor. The detection method was based on the modified protocol for bead-based immunoassays applying the combination of Luminex/xMAP technology and the ELISA protocol (Fig. 1a). An aliquot of the primary antibody-coated MBs complex against TF or ALB biomarkers was first diluted in diluent (1% BSA diluted in 1X PBS), and 50 μL of suspended beads was loaded to each well of a 96-well plate. The MBs were then separated and fixed from the supernatant by placing the plate on a magnet separator for 2 min and washed twice with diluent. Each time a multichannel pipette was used to carefully aspirate the supernatant from each well. Next, MBs were resuspended in the 50 μL bioreactor sample or standard biomarker solutions (0–16,000 ng/mL), and the mixtures were incubated for 30 min at room temperature on the plate shaker set at 800 rpm. Following the incubation, the plate was placed into the magnetic separator for bead separation. The supernatant was carefully aspirated from each well and the MBs were resuspended in 50 μL of diluent and washed twice. After the washing step, 50 μL of diluted biotinylated secondary antibody solution (1–10 μg/mL) was added to each well and mixed with antigen captured MBs followed by 30 min incubation on the plate shaker. MBs were then separated from the supernatant by the magnetic plate and rinsed twice with diluent. 50 μL of SA-HRP serial dilution (0.01–0.1 μg/mL) was loaded to each well, and the mixtures were incubated for additional 30 min on the plate shaker. Following incubation, the supernatant was removed from each well and 1:1 mixture of TMB and H_2_O_2_ solution was introduced to wells after the washing step. The plate was incubated for 15 min and the resulting solution was removed from the MBs and pipetted onto the EC microelectrode surface for detection. Current generated by the amount of oxidized TMB on the microelectrode was measured through amperometry method, which is proportional to the amount of target biomarker in the sample solution. To confirm the detection, an aliquot of supernatant was added to a new 96-well plate to be measured by the absorbance of 450 nm using a plate reader (BioTek, Winooski, VT, USA). In addition, the commercially available ELISA from Abcam was used to validate the developed bead-based immunosensing method.

### On-chip immunoassay measurement

In order to run on-chip electrochemical immunosensing, we made a microfluidic chip with built-in microvalves to replace the wells and create a platform which can do in-line automated and continuous detection. A neodymium magnet was positioned under the reaction chamber to capture and immobilize MBs. Beads suspension, samples, and assay solutions were introduced to the chip through the fluidic layer (red color) using the programmed N_2_ gas-actuated microvalves (green color) (Figs 1b  and S7d). All binding steps of the immunoassay were exerted at a flow rate of 1,000 μL/hr. The washing buffer was introduced into the microfluidic chip between each binding step to remove excess solution from the former step and minimize a background noise and false positive signals (Fig. S7d).

Prior to bead loading, channels were filled with the blocking solution via valve 12 and incubated for 30 min to avoid non-specific bindings. 65 μL of primary antibody-coupled MBs suspension was dispensed from the bead reservoir (2 × 10^4^ beads/mL) and loaded via valve 11 into the reaction chamber wherein approximately 1,250 MBs were immobilized by the magnet underneath the reaction chamber (Fig. S10a). During bead loading, valve 5 was open and both valves 4 and 6 stayed closed (Fig. S7d). To prevent bead sedimentation in the reservoir, MBs were agitated by a magnetic field generated around the reservoir. After loading, excessive MBs were washed out by washing buffer introduced from valve 10. Next, 150 μL aliquot of either a bioreactor sample or a standard biomarker solution (0–16,000 μg/mL) was loaded into the reaction chamber by opening valve 2. After 30-min incubation, 150 μL of 2.5 μg/mL secondary antibody and 1.0 μg/mL SA-HRP solutions were sequentially introduced to the reaction chamber by opening valves 14 and 13 respectively followed by a 30-min incubation at each step. After rinsing MBs with the washing buffer, 150 μL of TMB-H_2_O_2_ mixture was introduced to immobilized MBs by opening valve 3. Following 15 min incubation, the final product was moved to the detection chamber by opening valve 6, where the oxidized TMB from the enzymatic reaction was measured by the EC microelectrode. At the end of each replicate analysis, magnetic field was released and MBs were flushed out (Fig. S10b) by opening the outlet valve 5 followed by cleaning the chip with washing buffer and extracting the waste by opening outlet valves 7 and 8.

### Cell counts

Cell counts were performed with the hemocytometer method. GelMA samples containing hepatic spheroids were exposed to a 1.0 mg/mL collagenase II solution dissolved in 1X PBS and placed at 37 °C for 7 min until the hydrogel fully degraded. After adding culture media, spheroids were then treated with 1X trypsin for 5 min at 37 °C after which the cell suspension was centrifuged. The supernatant was discarded and the pellet was resuspended in the culture media for the cell count with a hemocytometer.

### Live/dead assay and microscopy

To assess the viability of the cells cultured inside the control and APAP treated bioreactors, LIVE/DEAD^®^ viability assay (Life Technologies) was performed at Days 1, 3, and 5. Fluorescence images were taken with a Zeiss Axio Observer D1 Fluorescence Microscope (Carl Zeiss, Thornwood, NY, USA). Analysis was implemented using the ImageJ software (National Institutes of Health, Bethesda, MD, USA).

### Statistical analysis

For data analysis, all quantified values were presented as mean ± standard error of the mean (SEM). All experiments were performed three times using independent replicates. Paired Student’s t-tests were concocted for statistical analysis. Statistically significant results were accepted at *P < 0.05, **P < 0.01, or ***P < 0.001. To determine the LOD of the biosensor, non-linear regression was applied to analyze the data. The LOD values were determined by the two standard deviations above the background level representing approximately 95% probability levels. A linear curve was fitted to the data using a regression analysis, to convert the signal value into the corresponding target biomarker concentration.

## Additional Information

**How to cite this article**: Riahi, R. *et al*. Automated microfluidic platform of bead-based electrochemical immunosensor integrated with bioreactor for continual monitoring of cell secreted biomarkers. *Sci. Rep*. **6**, 24598; doi: 10.1038/srep24598 (2016).

## Supplementary Material

Supplementary Information
