# Supplementary material for: Automated microfluidic platform of bead-based electrochemical immunosensor integrated with bioreactor for continual monitoring of cell secreted biomarkers
Source: Sci Rep. 2016 Apr 21;6:24598. doi: 10.1038/srep24598 (PMC4838915; doi:10.1038/srep24598)
Supplement: Supplementary Information [file srep24598-s1.pdf]

# Supplementary Information

## Automated microfluidic platform of bead-based electrochemical immunosensor integrated with bioreactor for continual monitoring of cell secreted biomarkers

Reza Riahi[1,2], Seyed Ali Mousavi Shaegh[1,2], Masoumeh Ghaderi[1,2], Yu Shrike Zhang[1,2,3], Su Ryon Shin[1,2,3], Julio Aleman[1,2], Solange Massa[1,2], Duckjin Kim[1,2], Mehmet Remzi Dokmeci[1,2,3], Ali Khademhosseini[1,2,3,4,5*]

[1]Harvard-MIT Division of Health Sciences and Technology, Massachusetts Institute of Technology, Cambridge, MA 02139, USA

[2]Biomaterials Innovation Research Center, Department of Medicine, Brigham and Women's Hospital, Harvard Medical School, Boston, MA 02139, USA

[3]Wyss Institute for Biologically Inspired Engineering, Harvard University, Boston, MA 02139, USA

[4]Department of Physics, King Abdulaziz University, Jeddah 21569, Saudi Arabia

[5]College of Animal Bioscience and Technology, Department of Bioindustrial Technologies, Konkuk University, Hwayang-dong, Kwangjin-gu, Seoul 143-701, Republic of Korea

[*] A. Khademhosseini, Corresponding-Author, E-mail: alik@bwh.harvard.edu

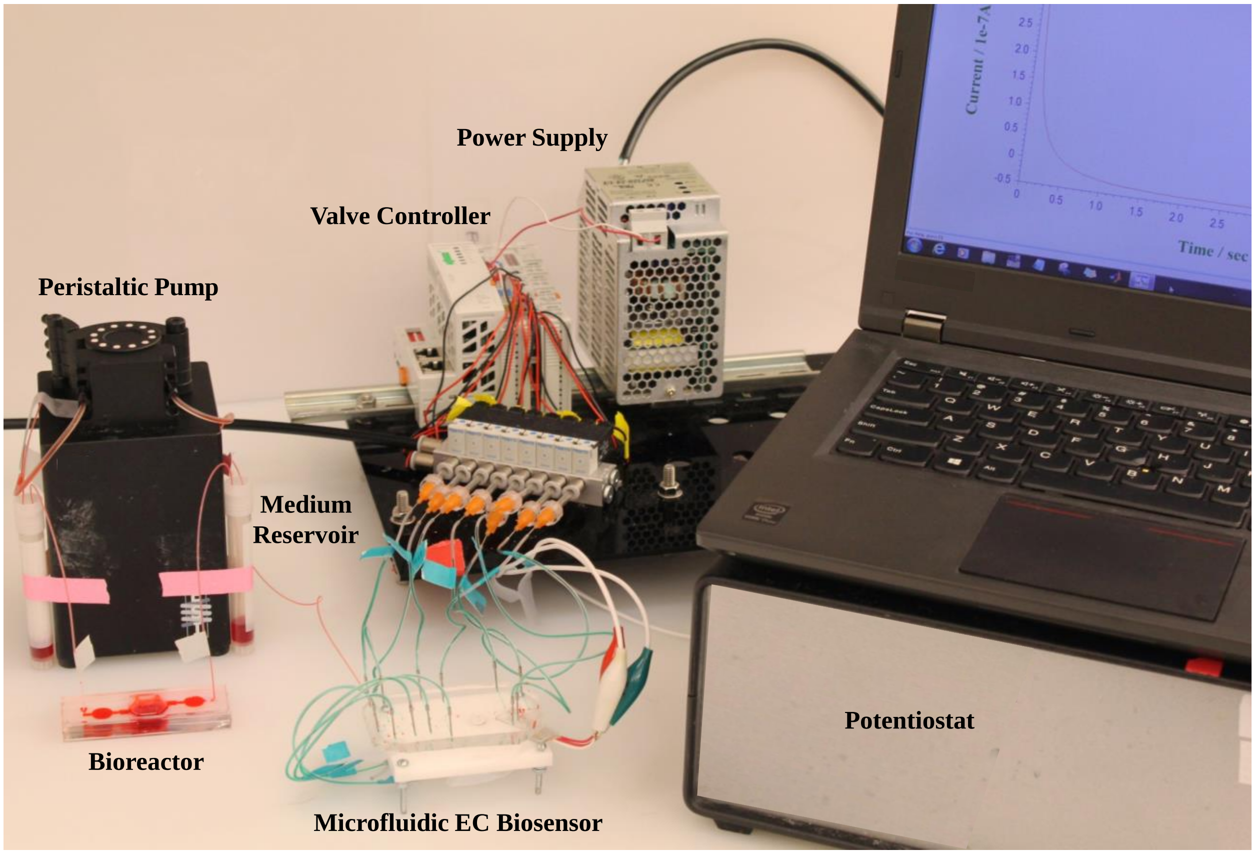


**Figure S1.** Photograph of the integrated EC immunosensing system for continual monitoring of the biomarkers secreted from the liver bioreactor.

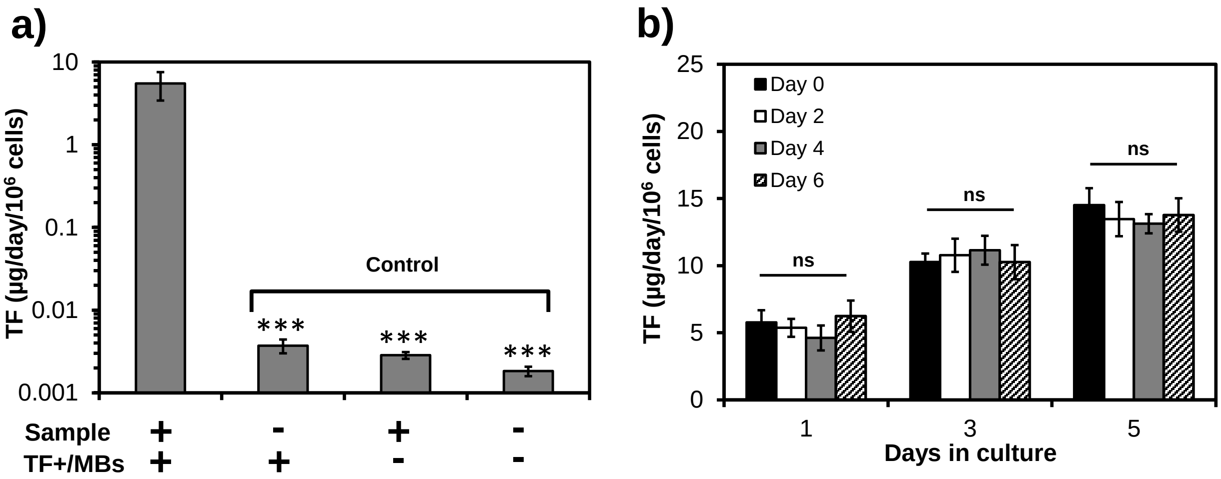


**Figure S2.** Off-chip specificity and stability of the developed EC immunosensing approach. a) Specificity of the EC immunosensing system in the presence and absence of TF+MBs and a bioreactor sample obtained from Day 1. b) Stability and reproducibility of the EC sensor by measuring Day 1, Day 3, and Day 5 samples from a single bioreactor at 4 individual days. Data are representative of three independent experiments (ns = not significant; ***, $P < 0.001$).

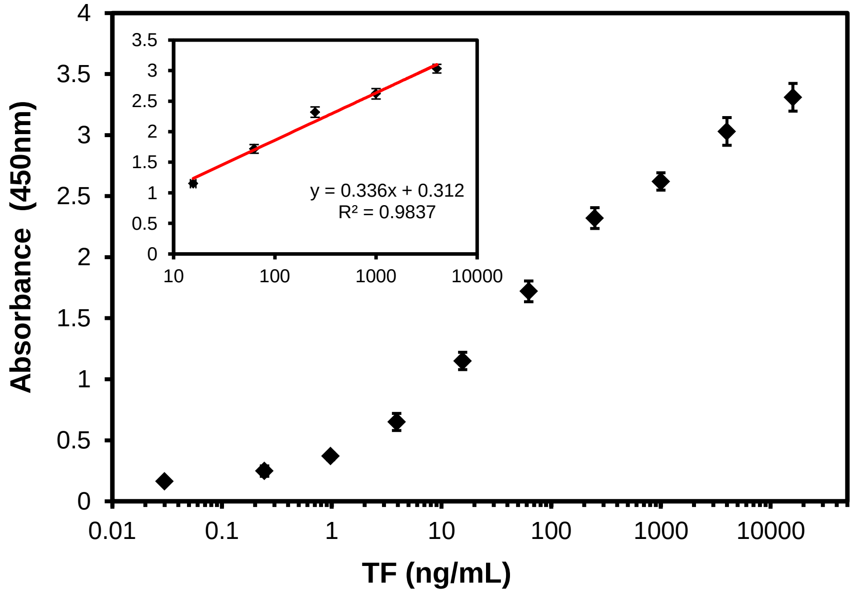


**Figure S3.** Standard curve for different concentration of standard TF solution obtained by ELISA using the plate reader. Insert is linear regression between 10-4000 ng/mL TF concentrations.

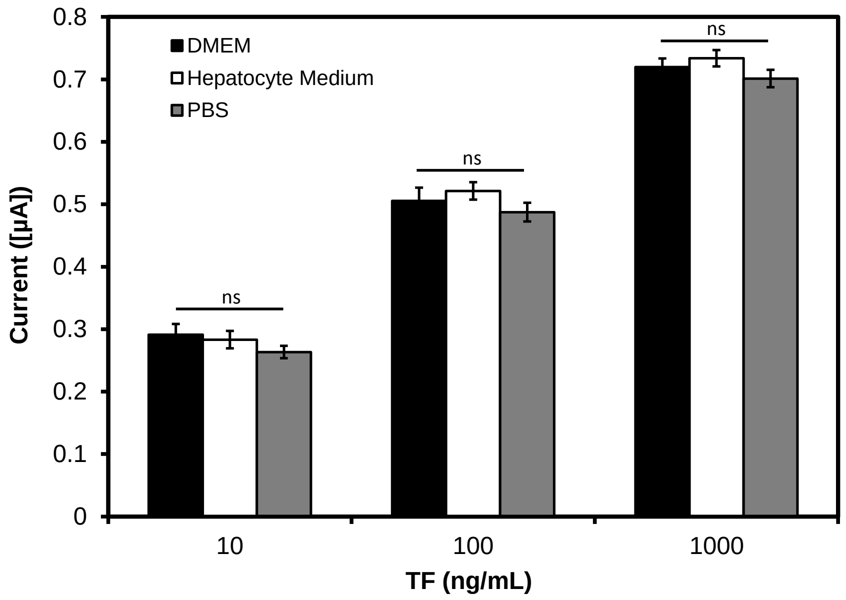


**Figure S4.** Effect of cell culture medium on EC measurement using different concentrations of standard TF solution. Data are representative of three independent experiments (ns=not significant).

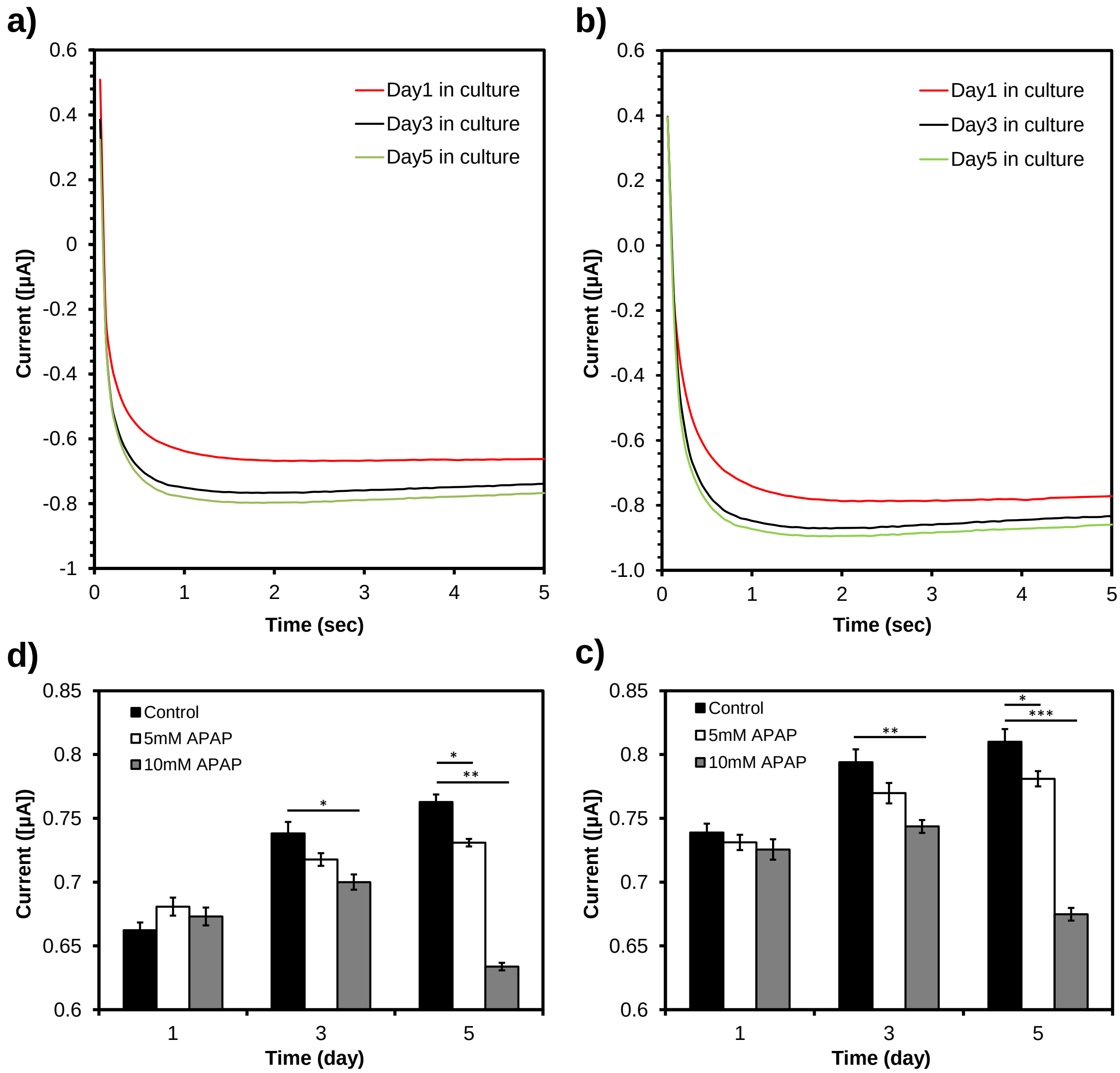


**Figure S5.** EC measurement of biomarkers secreted from primary hepatocytes cultured inside a bioreactor. a-b) Amperometry measurement at −100mV for the average concentration of TF (a) and ALB (b) biomarkers in culture medium at different days without drug treatment. c-d) Current signal corresponding to different TF (c) and ALB (d) concentrations obtained from liver media after the liver cells were treated with 5 mM and 10 mM APAP. Data are representative of three independent experiments (ns=not significant; *, P < 0.05; **, P < 0.01; ***, P < 0.001).

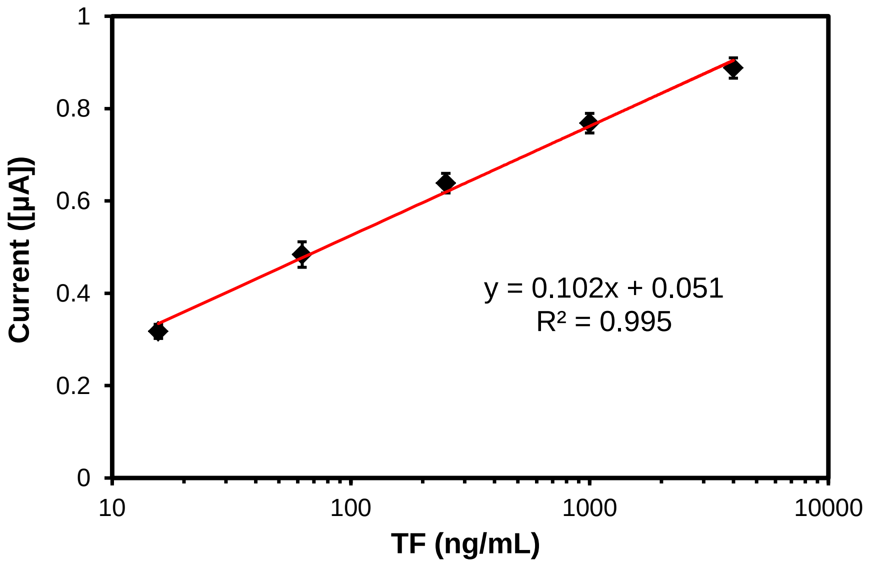


**Figure S6.** EC calibration curve of ALB biomarker obtained from standard ALB solution (15-4000 ng/mL) using the linear regression analysis.

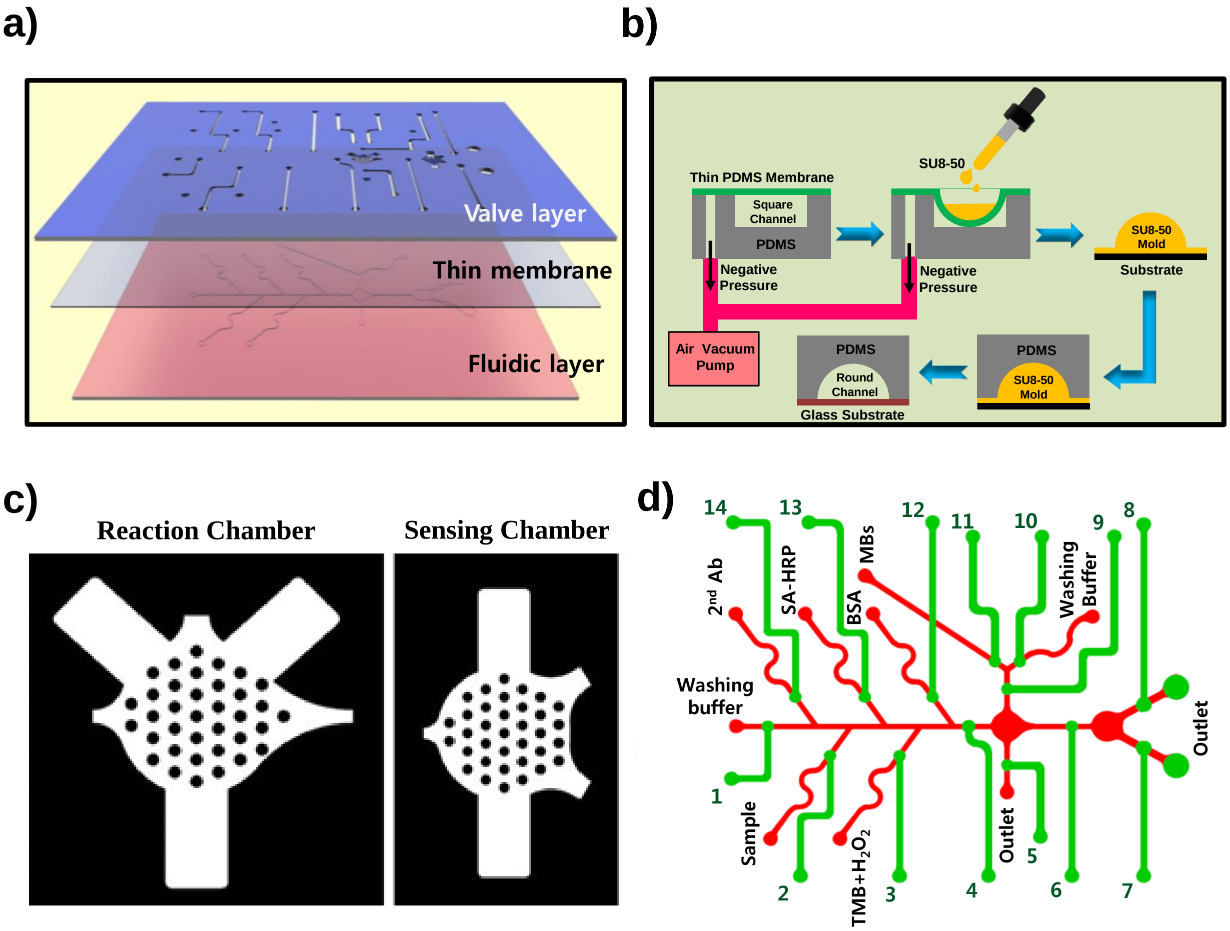


**Figure S7.** Design and fabrication of bead-based sensing chip. a) Schematic of the exploded multi-layer microfluidic chip comprising of a PDMS fluidic layer, the valve layer, and the thin PDMS membrane sandwiched in between. b) Fabrication process for the fluidic layer from the square to semi-circular cross-section using a thin PDMS membrane. c) Schematic of the fabricated bubble-trap consisting of a micropost array used for bubble removal from reaction and detection chambers. d) Design diagram of the microfluidic EC sensing chip where the fluidic layer is labeled in red and the valve layer in green.

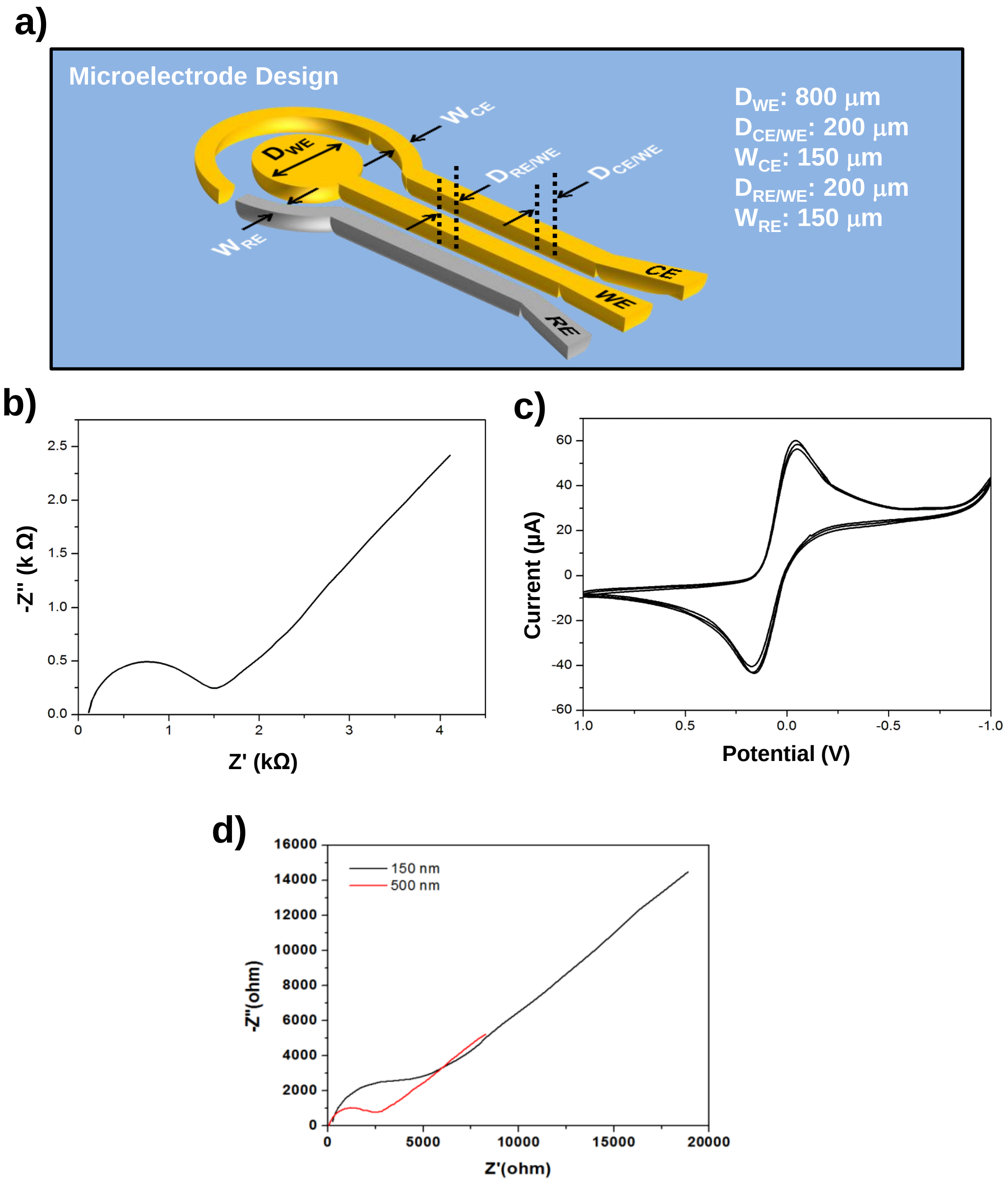


**Figure S8.** Characteristics of the three-electrode sensor. a) Design of the microelectrode with selected dimensions of widths (W) and distances (D) between counter electrode (CE) and working electrode (WE) as well as reference electrode (RE) and WE. b) Nyquist curve of the fabricated microelectrode in the presence of 50 mM $K_3[Fe(CN)_6]$ solution. c) Cyclic voltammetry (CV) of the microelectrode in the presence of 50 mM $K_3[Fe(CN)_6]$ solution performed at a scan rate of -100 mV/s with 10 repeated cycles. d) Nyquist curve of the microelectrodes with the 150 nm and 500 nm thick Au layer.

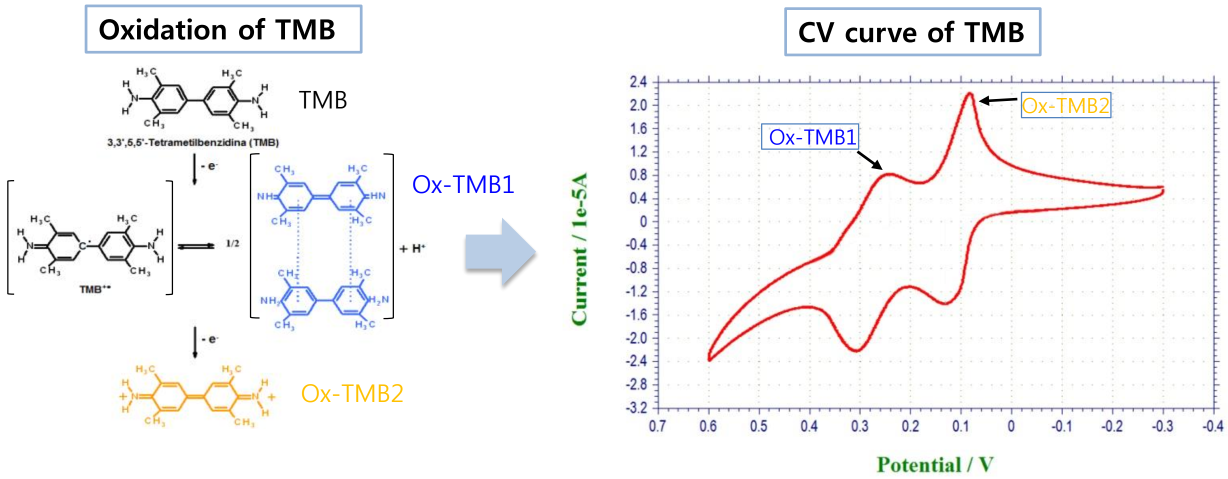


**Figure S9.** Working principle and CV curve showing the oxidation of TMB at different applied potentials to the EC sensor.

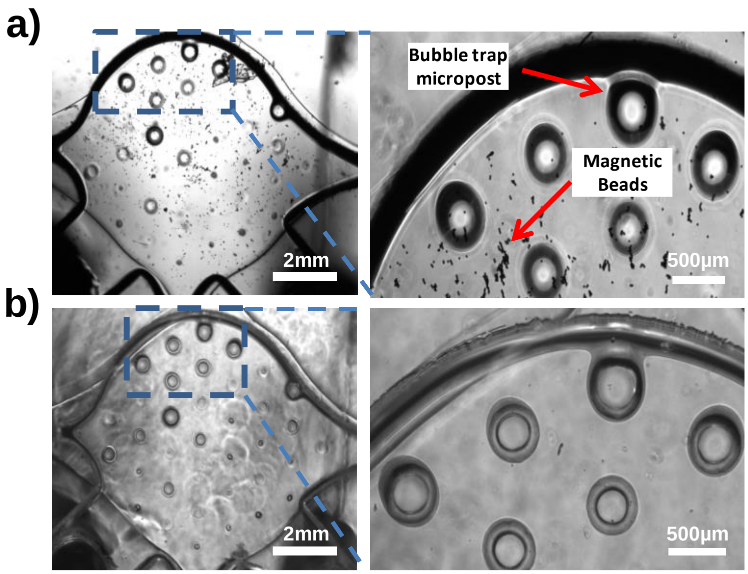


**Figure S10.** Photograph of the reaction chamber after: (a) immobilization of MBs and b) flushing out the MBs at the end of immunoassay process. Circular structures represent the bubble trap microposts located on the top surface of the reaction chamber.
